# Trajectory Optimization of Airport Surface Guidance Operations for Unmanned Guidance Vehicles

**DOI:** 10.3390/s26030931

**Published:** 2026-02-01

**Authors:** Tianping Sun, Kai Wang, Ke Tang, Dezhou Yuan, Xinping Zhu

**Affiliations:** 1 Air Traffc Management College, Civil Aviation Flight University of China, Chengdu 641419, China; 18137217056@163.com (T.S.); wkmfx@cafuc.edu.cn (K.W.); tangke@cafuc.edu.cn (K.T.); 2 School of Automotive Engineering, Liuzhou Polytechnic University, Liuzhou 545000, China; yuandezhou@lzpu.edu.cn

**Keywords:** airport movement area, unmanned guidance vehicles, trajectory planning, conflict prediction, conflict resolution

## Abstract

**Highlights:**

**What are the main findings?**

- A three-stage trajectory planning framework is proposed for airport surface unmanned guidance operations, which operates under operational safety constraints and integrates improved A* trajectory planning, time-window-based conflict prediction, and priority-driven conflict resolution.
- By incorporating speed-profile-based time calculation and spatiotemporal occupancy modeling of guidance vehicles and guidance units, the proposed method enables conflict-free trajectory generation while enhancing taxiing efficiency and reducing energy consumption.

**What are the implications of the main findings?**

- The results demonstrate that trajectory planning based on spatiotemporal state information—derived from speed profiles and time-window modeling—can effectively support safe and efficient unmanned guidance operations conducted by guidance vehicles and guidance units on airport surfaces.
- The proposed framework provides a practical decision-support framework for intelligent airport surface operations, supporting the deployment of electric unmanned guidance vehicles contributing to low-carbon and high-efficiency airport ground movement management.

**Abstract:**

Electric-powered unmanned guidance vehicles provide surface taxiing guidance for arriving and departing aircraft within the airport movement area, enabling sustained safety under complex operational conditions and improving overall operational efficiency, particularly under low-visibility scenarios. In this context, how to design scientifically rigorous operational trajectories for the three phases of unmanned guidance vehicle operations—dispatch, guidance, and recovery—remains an open and important research problem. This study proposes a three-stage trajectory-planning method for unmanned guidance vehicles, including initial trajectory planning, conflict prediction, and conflict resolution. First, the Guidance Unit—composed of the unmanned guidance vehicle and the guided aircraft—is defined, and a standard speed-profile design model is established for this unit. Then, considering airport operational-safety constraints, a conflict prediction algorithm for the guidance process is developed, which identifies potential conflicts in guidance trajectory planning based on time-window overlap analysis. Subsequently, under operational safety constraints, an optimization model aiming to minimize the maximum guidance time is formulated, and a trajectory planning algorithm for unmanned guidance vehicles based on the improved A* algorithm is designed to generate conflict-free operational trajectories. Finally, a simulation study is conducted using a major airport in Southwest China as a case study. The results show that (1) the speed-profile design and airport operational-rule constraints affect the operational trajectories of unmanned guidance vehicles; (2) the proposed algorithm enables coordinated planning of both speed control and path selection, thereby improving overall operational efficiency by 43.65% compared with conventional operations, while ensuring conflict-free airport surface taxiing, due to the adoption of an improved A* trajectory-planning algorithm for unmanned guidance vehicles; (3) under the electric-powered guidance-vehicle scheme proposed in this study, the method achieves a 34.52% reduction in total energy consumption during the guidance phase compared with traditional Follow-Me guidance, enabling the simultaneous optimization of operational efficiency and energy consumption.

## 1. Introduction

The airport movement area contains numerous active targets, with high levels of aircraft and ground-vehicle activity and dense traffic. In large and complex airports in particular, the energy consumption is high, and the operational load is heavy, and aircraft may easily stray onto incorrect taxiing routes, thereby disrupting normal operations or even causing runway incursions and other safety-critical incidents. Airport unmanned equipment refers to a new generation of airport operational devices that operate within the airport movement area by integrating advanced sensors, controllers, and other embedded components, and employing artificial intelligence technologies to enable autonomous functions such as perception in complex environments, intelligent decision-making, and cooperative control [1]. With the maturation of unmanned-driving technologies, electric-powered unmanned guidance vehicles can provide surface taxiing guidance for arriving and departing aircraft, ensuring sustained operational safety under high-density and complex operational conditions and enabling highly efficient, low energy consumption, and environmentally friendly airport surface operations. However, airport operations are safety-critical in nature, and how to design an efficient, low energy consumption, and scientifically sound trajectory-planning scheme for such operations remains a topic worthy of in-depth investigation [2].

Extensive research has been conducted on aircraft surface taxiing trajectory planning. Among these studies, Zhang T. et al. [3] proposed a conflict-free trajectory planning model based on a time-driven framework, where conflict prediction and avoidance are achieved through a refined taxiway topology modeling and segmented temporal constraints. Subsequently, Zhang T. et al. [4] integrated an improved A* algorithm with time-window constraints, significantly enhancing trajectory smoothness and operational safety under high-density taxiing scenarios. Zhang T. et al. [5] adopted a rolling time-domain computation method to formulate an integer linear programming model for conflict-free flight trajectories and validated the proposed approach under multiple traffic-density scenarios. Pazhooh et al. [6] proposed a continuous-time mixed-integer linear programming (MILP) model that simultaneously addresses path selection, spatial-blocking constraints, time-window considerations, and resource-conflict issues within a unified framework. In related studies, conflict prediction and resolution have been extensively investigated, as the generation of conflict-free operational trajectories is a core research focus [7]. For example, Zhou et al. [8] proposed a conflict-detection and local-adjustment method based on spatial protection zones and time-occupancy intervals, enabling dynamic avoidance of taxiing conflicts. Wang et al. [9] proposed a chance-constrained planning model under taxi-time constraints, achieving safe and efficient trajectory generation. Yuan et al. [1] developed a conflict-prediction model for unmanned Follow-Me guidance, embedding spatiotemporal safety constraints into the trajectory-generation process; Zhuge et al. [10] proposed a multi-level conflict-warning model based on control commands and ADS-B data, which identifies potential conflicts in advance by predicting the probability distribution of taxiing paths. Sui et al. [11] integrated prior knowledge with Monte Carlo Tree Search to construct a conflict-resolution framework applicable to taxiway intersections, achieving a conflict-resolution success rate of approximately 96.8% across various typical conflict scenarios.

In addition, with respect to the joint optimization of trajectories and energy consumption, Lipp et al. [12] provided a convexified solution for minimum-time speed optimization along fixed trajectories, offering a theoretical foundation for the three-phase speed profile of “acceleration–cruise–deceleration.” Zhang et al. [13] proposed an online speed-profile generation method that produces energy-efficient taxiing speed curves under time-window and energy-consumption constraints. Weiszer et al. [14] developed a multi-objective ground-operations optimization framework that integrates operational efficiency and environmental metrics within the trajectory-planning layer. Evertse et al. [15] established a real-time ground-trajectory optimization model aimed at minimizing emissions, achieving a unified treatment of conflict resolvability and green operations. Li et al. [16] explored low-emission taxiing path generation methods by incorporating turning frequency, average taxiing speed, and fuel intensity into a joint optimization framework.

In summary, the commonly used modeling approaches in existing studies include mixed-integer linear programming, graph-theoretical methods, convex optimization, and combinatorial optimization, while the frequently employed algorithms comprise exhaustive searches, heuristic searches, genetic algorithms, and particle swarm optimization. Despite these advances, existing research still exhibits several limitations. First, time-window constraints and trajectory replanning for operational path adjustments are often considered separately from energy-consumption optimization, and a unified framework for joint optimization remains lacking. Second, the design of speed-profile models and energy consumption models has not yet been fully aligned with the actual operational requirements of unmanned guidance at airports, and related optimization studies remain limited. Finally, current research provides insufficient holistic consideration of practical future unmanned guidance scenarios, particularly due to the lack of effective integration of trajectory planning into existing scheduling systems, resulting in limited system-level coordination and validation.

This study focuses on the airport-surface taxiing process of arriving and departing aircraft and designs a guidance operation workflow based on unmanned guidance vehicles. By considering surface-traffic conflict prediction and resolution and operational efficiency requirements, a trajectory planning algorithm for guidance operations is developed.

## 2. Problem Analysis

**Figure 1:**
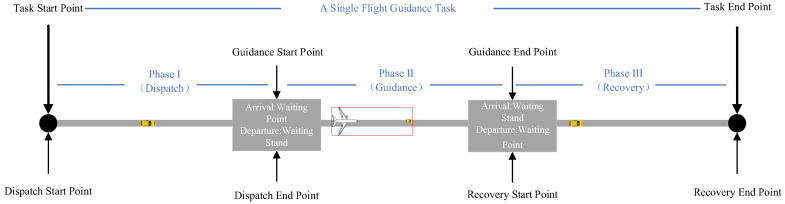
Abstraction of the guidance process for arriving and departing aircraft.

The implementation of aircraft surface taxiing operations assisted by unmanned guidance vehicles consists of three distinct phases: dispatch, guidance, and recovery. As illustrated in Figure 1, the dispatch phase refers to the process in which an unmanned guidance vehicle, after receiving its task assignment, departs from its designated parking stand and travels across the airport surface to reach the position of the aircraft waiting to be guided by the unmanned guidance vehicle. The guidance phase refers to the process in which the unmanned guidance vehicle escorts an arriving or departing aircraft from its taxi-start position to the taxi-end position along the airport surface taxiway network. The recovery phase refers to the process in which the unmanned guidance vehicle, after completing the guidance task, returns to its designated parking stand. To simplify the problem, the following assumptions are made:

1. During the dispatch and recovery phases, the unmanned guidance vehicle operates entirely within the airport surface taxiway network.
2. During the guidance phase, the unmanned guidance vehicle and the guided aircraft are regarded as a single kinematic entity, hereinafter referred to as the “Guidance Unit.” Accordingly, the airport surface trajectory planning for unmanned guidance primarily aims to assign appropriate unmanned guidance vehicles to arriving and departing flights within a given time horizon, and to determine the corresponding travel routes and the passage times at key waypoints for each unmanned guidance vehicle or guidance unit, so as to ensure the minimum taxiing time and conflict-free surface operations.

**Figure 2:**
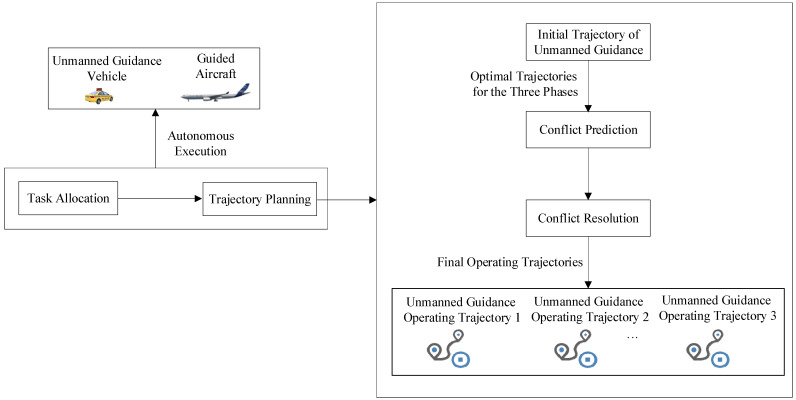
Implementation framework of unmanned guidance trajectory planning.

For aircraft that successively enter surface operations according to their flight schedules, as illustrated in Figure 2, airport surface trajectory planning for unmanned guidance consists of three major modules: initial trajectory planning, conflict prediction, and conflict resolution [17]. Among them, the initial trajectory planning refers to the process of generating the initial optimal trajectory for the dispatch, guidance, and recovery phases of an aircraft’s unmanned guidance operation, subject to specific safety and operational constraints; conflict prediction refers to the process of evaluating, based on the predetermined trajectories of existing active targets on the airport surface (including unmanned guidance vehicles or guidance units), whether a newly introduced unmanned guidance vehicle or a newly formed guidance unit will have potential conflicts with those existing active targets when taxiing along its initial optimal trajectory; conflict resolution refers to the process of applying appropriate resolution strategies, once conflicts are identified through conflict prediction, to adjust the trajectories of the relevant active targets so as to eliminate the predicted conflicts and ultimately achieve conflict-free operations with the minimum taxiing time throughout the entire unmanned guidance process.

## 3. Unmanned Guidance Unit

In the process of aircraft surface taxiing operations implemented with unmanned guidance vehicles, the overall procedure consists of three phases: dispatch, guidance, and recovery. Among them, the guidance phase involves cooperative taxiing, in which the guided aircraft operates under the pilot’s control while following the unmanned guidance vehicle. In this phase, due to the differences in cockpit heights among various aircraft types, the unmanned guidance vehicle and the guided aircraft are regarded as a single kinematic entity—referred to as the “Guidance Unit”—to maintain a reasonable and relatively stable following distance while coordinating with the interactions of other active targets on the airport surface. Accordingly, the following definition is introduced to describe the “Guidance Unit” [18].

**Figure 3:**
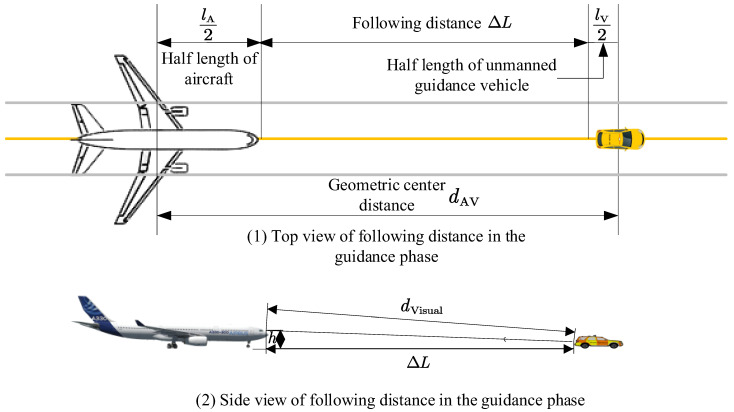
Guidance Unit.

**Definition** **1.** *Guidance Unit. During the guidance phase, a following relationship is continuously maintained between the unmanned guidance vehicle and the guided aircraft. The unmanned guidance vehicle and the guided aircraft are therefore defined as a surface operational “Guidance Unit” in this scenario, as illustrated in Figure 3.*

As shown in Figure 3, within the guidance unit, the longitudinal distance from the aircraft nose to the rear of the unmanned guidance vehicle is defined as $\Delta L=d_{AV}-\frac{l_{A}}{2}-\frac{l_{V}}{2}$, where $l_{A}$ and $l_{V}$ denote the lengths of the aircraft and the unmanned guidance vehicle, respectively, and $d_{AV}=\sqrt{{\left(x_{A}-x_{V}\right)}^{2}+{\left(y_{A}-y_{V}\right)}^{2}}$ represents the geometric distance between their centroids. Meanwhile, during the guided taxiing process, the pilot’s visual perception should be taken into account, and a comprehensive evaluation is required to determine a safe following distance for each aircraft type. The pilot’s visual distance $d_{visual}$ and the guidance following distance ΔL are related as follows: $d_{visual}=\sqrt{h^{2}+\Delta L^{2}}$; the following distance ΔL can be determined with reference to the current manned Follow-Me operations on the airport surface, and its average value is approximately 60 m, following the setting adopted in Yuan et al. [1], where a similar unmanned Follow-Me guidance scenario on the airport surface was investigated. This value reflects typical operational safety requirements under airport taxiing conditions and ensures consistency with existing studies.

**Definition** **2.** *Guidance Trajectory. The guidance trajectory is defined as the time-stamped path of the geometric center of the unmanned guidance vehicle or the guidance unit across key positions on the airport surface.*

## 4. Mathematical Description of the Unmanned Guidance Trajectory Planning Problem

### 4.1. Airport Runway and Taxiway Network

Let $G_{X}$ denote the runway–taxiway system network within the airport movement area, which consists of runways, taxiways, stands, and parking positions. This network can be regarded as either a topological traffic network or a physical network of the runway–taxiway system. The runway–taxiway system is modeled as a directed graph $G_{X}=\left(N_{R}\cup N_{X}\cup N_{V}\cup N_{W},E_{X}\right)$, where $r_{rwy\_entry}\in N_{R}$, $r_{G}\in N_{R}$ denote the runway entry, runway exit, and aircraft stand nodes, respectively; $N_{X}$ represents the set of taxiway intersections, $N_{V}$ the set of parking positions, and $N_{W}$ the set of waiting points near stands or runway exits, such that $W_{gate}\in N_{W}$, $W_{rwy\_exit}\in N_{W}$. The connections among these nodes are established through the following directed edges: $E_{X}^{R}\subseteq N_{X}\times N_{R}$, $E_{X}^{V}\subseteq N_{X}\times N_{V}$, $E_{X}^{W}\subseteq N_{X}\times N_{W}$.

Let $L_{X}:E_{X}\cup E_{X}^{R}\cup E_{X}^{V}\cup E_{X}^{W}\to {\mathbb{R}}^{+}$ denote the length of each edge in the network $G_{X}$ and $v_{X}:E_{X}\cup E_{X}^{R}\cup E_{X}^{V}\cup E_{X}^{W}\to {\mathbb{R}}^{+}$ denote the operating taxi speed adopted on each edge.

As previously stated, it is assumed that the entire operation of the unmanned guidance vehicle is completed solely within the runway–taxiway system $G_{X}$. For clarity and consistency of scientific notation, the definitions and units of all major symbols are summarized in Appendix A.

### 4.2. Description of the Unmanned Guidance Process

Let the set of arriving and departing flight guidance tasks be denoted by B. Let $B^{arr}$ denote the set of arriving flights, and $B^{dep}$ denote the set of departing flights. Accordingly, $B=B^{arr}\cup B^{dep}$, with $B^{arr}\cap B^{dep}=\emptyset$. Each flight b∈B corresponds to a guidance task, which consists of three phases: dispatch, guidance, and recovery [19]. Let F denote the set of arriving and departing flights considered in the airport surface operation and let f∈F represent a specific flight.

The three phases mentioned above can be represented by a tuple $b=\left(f,\varsigma ,p,d,t\right)$, where the flight f∈F contains the corresponding attributes such as arrival or departure type, aircraft model, and the initial taxiing start time; where ς=1,2,3 represent the dispatch, guidance, and recovery phases, respectively; the current phase starts from node p∈P, $p:I\to N_{R}\cup N_{V}\cup N_{W}$, and ends at node d∈D, $d:I\to N_{R}\cup N_{V}\cup N_{W}$; the starting time of the current phase is denoted as t∈T, t:I→T, where I denotes the index set of trajectory segments within the current phase.

#### 4.2.1. Guidance Process for Arriving Flights

**Figure 4:**
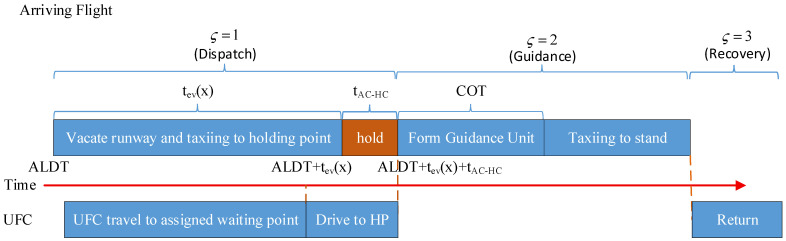
Time-sequence diagram of the guidance process for arriving flights.

As illustrated in Figure 4, for the arriving flight guidance process, after receiving its assigned task, the unmanned guidance vehicle proceeds to the vicinity of the taxiway exit of the assigned arriving flight $f_{arr}\in F$ during the dispatch phase $\left(\varsigma =1\right)$ from its designated parking stand and waits there; when the arriving flight leaves the runway and reaches the corresponding waiting point $r_{rwy\_exit}\in N_{W}$, the unmanned guidance vehicle drives to the vicinity of this waiting point and forms a guidance unit with the aircraft, with a coordination time denoted as COT.

During the guidance phase $\left(\varsigma =2\right)$, the guidance unit taxis along the planned trajectory to the assigned stand $r_{G}\in N_{R}$ of the arriving flight, and it is dissolved near the corresponding waiting point $W_{gate}\in N_{W}$ adjacent to that stand.

During the recovery phase $\left(\varsigma =3\right)$, the unmanned guidance vehicle travels through the taxiway system to its parking position $N_{V}$ or to the location corresponding to the next assigned guidance task.

#### 4.2.2. Guidance Process for Departing Flights

**Figure 5:**
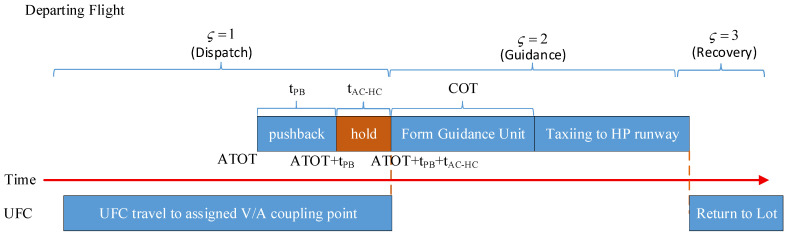
Time-sequence diagram of the guidance process for departing flights.

Similarly, as illustrated in Figure 5, for the departing flight guidance process, after receiving its assigned task, the unmanned guidance vehicle proceeds from its designated parking stand to the waiting point $W_{gate}\in N_{W}$ located near the assigned stand of the departing flight $f_{dep}\in F$ during the dispatch phase $\left(\varsigma =1\right)$; after the aircraft has completed the pushback operation from the stand, the unmanned guidance vehicle forms a guidance unit with the aircraft, with a coordination time denoted as COT; during the guidance phase $\left(\varsigma =2\right)$, the guidance unit taxis along the planned trajectory to the taxiway point $r_{rwy-entry}\in N_{R}$ located near the assigned departure runway, where it is dissolved, and the aircraft subsequently completes the take-off according to air traffic control instructions; during the recovery phase $\left(\varsigma =3\right)$, the unmanned guidance vehicle travels through the taxiway system to its parking position $N_{V}$ or to the location corresponding to the next assigned guidance task.

### 4.3. Speed Profile Design for Unmanned Guidance Operations

During surface operations, the unmanned guidance vehicle or the guidance unit is subject to corresponding speed limits on straight and curved segments of the taxiway, resulting in acceleration and deceleration processes accordingly. In addition, to avoid potential conflicts, the unmanned guidance vehicle or the guidance unit may perform waiting operations at appropriate locations on the airport surface when necessary. Therefore, to achieve refined trajectory planning for unmanned guidance operations, it is necessary to design the corresponding speed profile [20]. For simplification, the unmanned guidance vehicle is assumed to travel at a constant speed throughout the dispatch and recovery phases (neglecting acceleration and deceleration processes), while the speed profile design of the guidance unit during the guidance phase is emphasized. This modeling choice is adopted to avoid introducing unnecessary complexity in the dispatch and recovery phases, which are mainly included to ensure the completeness of the unmanned guidance operation workflow. Since the dominant characteristics of trajectory feasibility, spatiotemporal conflict interaction, and operational timing are primarily determined by the guidance phase, the constant speed assumption in the dispatch and recovery phases does not affect the main conclusions of this study. In contrast, the guidance phase involves the coupled operation of the unmanned guidance vehicle and the guided aircraft, where speed regulation and motion smoothness are critical for ensuring operational safety and feasibility. Therefore, an explicit acceleration–cruise–deceleration speed-profile model is employed in the guidance phase to characterize the spatiotemporal evolution of guidance operations.

During the taxiing process of the guidance unit, the curve taxiing speed is set as $v_{c}=5\;m/s$, the straight-line taxiing speed as $v_{s}$ (with a maximum value of $v_{\max}=10\;m/s$), the acceleration as $a_{cc}{=2\;m/s}^{2}$, and the deceleration as $a_{dc}=-1.5\;{m/s}^{2}$ [21]. The speed-profile parameters are referenced from typical taxiing-speed magnitudes in the literature and parameterized for unmanned guidance vehicle operations [22], forming a unified kinematic basis for the entire trajectory planning process.

The acceleration and deceleration processes of the guidance unit occur on the straight segments either before entering a curve or after leaving a curve.

For the taxiing process on consecutive straight segments, the acceleration and deceleration behaviors follow the same rule. For ease of expression, a series of consecutive straight segments along the path is defined as a straight-line unit.

**Figure 6:**
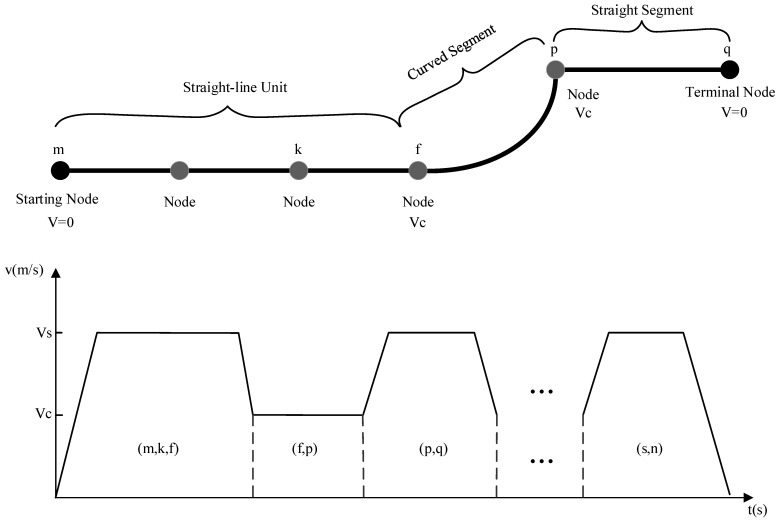
Example of the speed profile of the Guidance Unit.

For the taxiing process of the guidance unit along a given path $r=\left(m,k,f,p,\ldots ,s,n\right)$, the speed adjustment curve is illustrated in Figure 6. It is assumed that the acceleration and deceleration of the aircraft remain constant, the initial segment mk and the terminal segment sn are both straight segments, and the straight segments (straight-line units) and curves along the path r appear alternately.

For a given taxiing path, multiple speed profile curves can be generated by configuring different maximum taxiing speeds on each straight segment or straight-line unit, where each segment or unit is divided into three phases: acceleration, constant-speed, and deceleration. The specific speed profile can be determined based on the corresponding variables $a_{cc}$, $d_{1}$, $a_{dc}$, $d_{3}$, $d_{2}$ [23], where $a_{cc}$ denotes the acceleration adopted after entering a straight segment or a straight-line unit, and $d_{1}$ represents the acceleration distance; $a_{dc}$ represents the maximum deceleration applied before completing the movement on a straight segment or a straight-line unit; $d_{3}$ represents the deceleration distance; $d_{2}$ represents the constant-speed distance. The specific determination process is as follows:

Based on the above analysis, for the guidance unit, multiple distinct speed profile curves can be generated on straight segments or straight-line units according to different speed configurations. Therefore, three independent variables, $v_{s}$, $d_{1}$, and $d_{3}$, completely define a unique speed curve for a straight-line unit. However, the variables $v_{s}$, $d_{1}$, and $d_{3}$ must satisfy the physical constraints to ensure feasibility, and the basic parameter selection should satisfy:

Firstly, the definition of $v_{s}$ is given as follows. The upper bound of $v_{s}$ is equal to the maximum speed $v_{\max}$, while the lower bound $v_{profile}$ corresponds to the case in which the guidance unit accelerates within the straight segment or straight-line unit and must ensure that the terminal node of the segment or unit can reach the curve speed $v_{c}$:

(1) $$v_{s}=\min\left(v_{profile},v_{\max}\right)$$

(2) $$v_{profile}=\sqrt{\frac{2a_{cc}d+v_{in}^{2}+v_{c}^{2}}{2}}$$

In the equation, $v_{profile}$ represents the maximum speed of the straight segment or straight-line unit, a denotes the magnitude of acceleration, d represents the length of the straight segment or straight-line unit, and $v_{in}$ denotes the initial speed when entering the straight segment or straight-line unit.

Secondly, after $v_{s}$ is determined, the distance of the first stage $d_{1}$ can be calculated as follows:

(3) $$d_{1}=\frac{v_{s}^{2}-v_{in}^{2}}{2a_{cc}}$$

Next, after $v_{s}$ is determined, the distance of the third stage $d_{3}$ can be calculated as follows:

(4) $$d_{3}=\frac{v_{s}^{2}-v_{c}^{2}}{2a_{dc}}$$

The algorithm is presented as follows (Algorithm 1):
**Algorithm 1** Speed profile computation for unmanned guidance unit**Input:** Path of segments $\left\{Seg,Seg_{2},\ldots ,Seg_{n}\right\}$,   $\mathrm{where}\;Seg.type\in \left\{Straight,Curve\right\}$;   Segment parameters (length d, $\mathrm{arc}\;\mathrm{length}\;l_{c}$, $\mathrm{entry}\;\mathrm{speed}\;v_{in}$, $\mathrm{exit}\;\mathrm{speed}\;v_{c}$, $\mathrm{maximum}\;\mathrm{speed}\;v_{\max}$, $\mathrm{acceleration}\;a_{cc}$, $\mathrm{deceleration}\;a_{dc}$); **Output:** Speed profile set $\left\{v_{s},d_{1},d_{2},d_{3}\right\}$$\;\mathrm{for}\;\mathrm{straight}\;\mathrm{segments}\;\mathrm{and}\;\left\{v_{c},l_{c}\right\}$ for curves.    $1:\;\mathrm{for}\;\mathrm{each}\;\mathrm{segment}\;Seg\in \left\{Seg,Seg_{2},\ldots ,Seg_{n}\right\}$ **do**    2:  if Seg.type=Straight **then**    3:   if previous segment is Cruve$\;\mathrm{then}\;v_{in}\leftarrow v_{c}$;    4:   if next segment is Cruve$\;\mathrm{then}\;v_{c}\leftarrow v_{c}$;    $5:\;\;\;v_{profile}$← **Formula (2)**; $v_{s}$←**Formula (1)**;    $6:\;\;\;d_{1}$← **Formula (3)**; $d_{3}$←**Formula (4)**;    $7:\;\;\;\;\mathrm{if}\;d_{2}>0$$\;\mathrm{then}\;\mathrm{type}\leftarrow \mathrm{trapezoidal};\;\mathrm{else}\;\mathrm{type}\leftarrow \mathrm{triangular}\;\mathrm{and}\;\mathrm{recalc}\;\left(v_{s},d_{1},d_{3}\right)$;    $8:\;\;\;\;\mathrm{store}\;\left(v_{s},d_{1},d_{2},d_{3},type\right)$;    9: else if Seg.type=Curve **then**    $10:\;\;\;\;v_{c}$←constant value;    $11:\;\;\;\;\mathrm{store}\;\left\{v_{c},l_{c}\right\}$;    12: **end if**    13: **end for**    14: return complete speed profile for all segments.

### 4.4. Energy Consumption Model for Unmanned Guidance

To quantify the energy consumption of unmanned guidance during the airport surface taxiing operations, an energy consumption calculation model for unmanned guidance is established [24]. The energy consumption of the unmanned guidance vehicle is analyzed in the dispatch and recovery phases, while the energy consumption of the guidance unit is analyzed in the guidance phase [25]. The energy consumption in the guidance phase is established based on the three-phase speed profile model. During the taxiing operations, the unmanned guidance vehicle needs to overcome rolling resistance, aerodynamic drag, and the inertial force generated by its own acceleration [26]. Therefore, its instantaneous traction power can be expressed as follows:

(5) $$P\left(v,a\right)=\frac{\left(ma+c_{r}mg+\frac{1}{2}\rho C_{d}Av^{2}\right)v}{\eta }$$

In the equation, m denotes the equivalent mass of the unmanned guidance vehicle, g represents the gravitational acceleration, $c_{r}$ is the rolling resistance coefficient, ρ is the air density, $C_{d}A$ denotes the product of the aerodynamic drag coefficient and frontal area, and η represents the system efficiency. Equation (5) indicates that the traction power of the unmanned guidance vehicle consists of three components: the acceleration power, the power to overcome rolling resistance, and the aerodynamic drag power [27].

Energy consumption calculation under the given speed profile:

(6) $$E^{v}=\left\{\begin{array}{cc} \int_{v_{1}}^{v_{2}}\frac{p\left(v,a\right)}{a}dv & \mathrm{if}\;v_{1}\neq v_{2}\\ p\left(v,a\right)\frac{d}{v} & \mathrm{if}\;v_{1}=v_{2} \end{array}\right.$$

In the equation, $v_{1}$ represents the entry speed of the segment, $v_{2}$ denotes the exit speed, a represents the corresponding acceleration or deceleration, and d denotes the segment length. The unmanned guidance vehicle and the guidance unit maintain a constant speed when moving along curved segments, hence $v_{1}=v_{2}=v_{c}$. For the straight taxiway segment or straight-line unit, the parameters are defined as follows: during the acceleration phase, $v_{1}=v_{in}$, $v_{2}=v_{s}$, and $a=a_{cc}$; during the constant-speed phase, $v_{1}=v_{2}=v_{s}$; and during the deceleration phase, $v_{1}=v_{s}$, $v_{2}=v_{c}$, and $a=a_{dc}$.

The taxiing energy consumption of the aircraft during the guidance phase is estimated based on the aircraft type and taxiing time, and can therefore be expressed as follows [28]:

(7) $$E^{f}={\dot{m}}_{taxi}\times t_{seg}\times LHV$$

Therefore, the energy consumption of the guidance unit is the sum of the energy consumption of the unmanned guidance vehicle and that of the guided aircraft:

(8) $$E=E^{v}+E^{f}$$

## 5. Trajectory Planning Model for Unmanned Guidance

**Figure 7:**
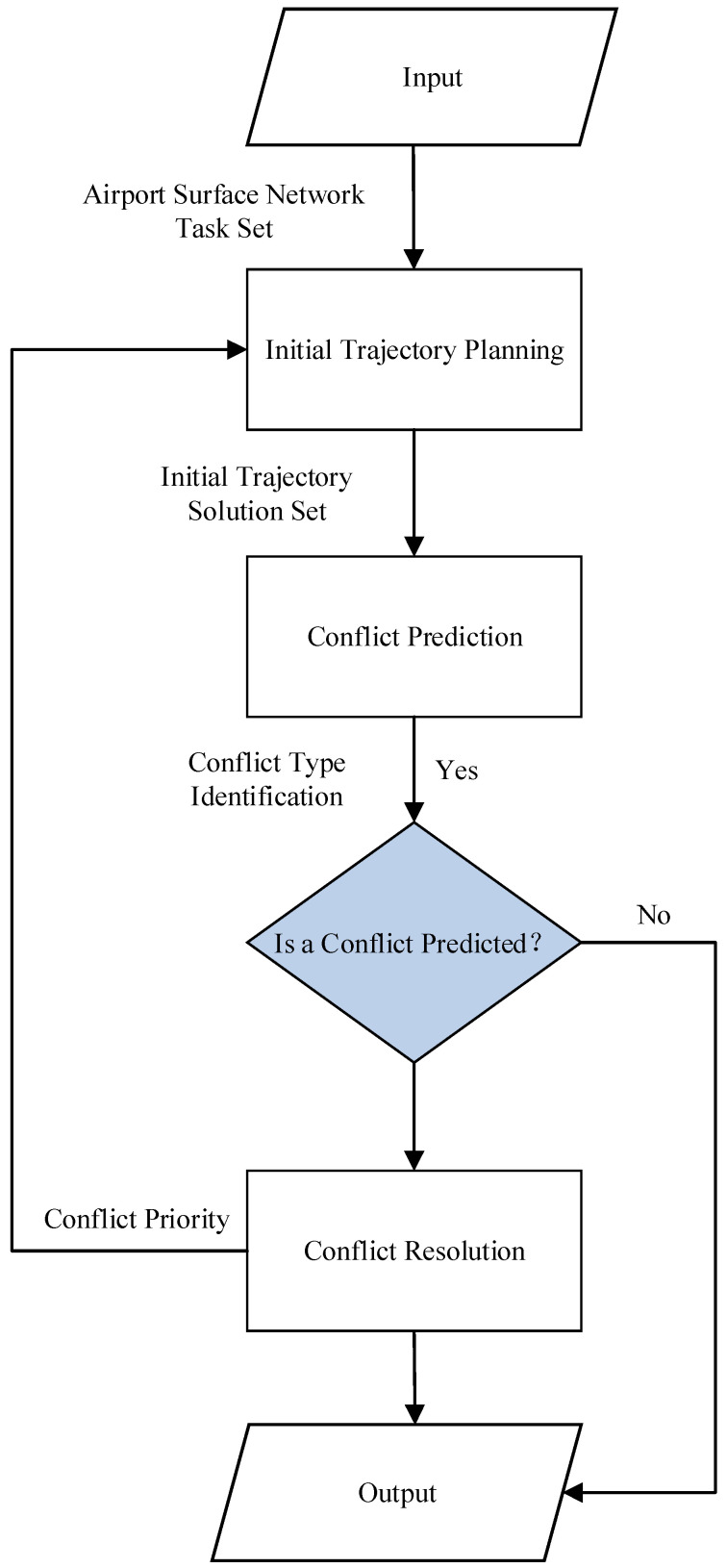
Framework of the unmanned guidance trajectory planning model.

The unmanned guidance trajectory planning consists of three modules: initial trajectory planning, conflict prediction, and conflict resolution. As shown in Figure 7, the initial trajectory planning module is responsible for generating the shortest initial operational trajectory and producing the initial trajectory solution set. The conflict prediction module is used to identify potential conflicts within the initial trajectory solution set and to determine the feasibility of the trajectory solutions. The conflict resolution module is used to eliminate potential conflicts and to regenerate the trajectory solution set for conflicting trajectories. Finally, the feasible solution set is integrated to generate the optimal trajectory solution.

The algorithm is presented as follows (Algorithm 2):
**Algorithm 2** Trajectory planning for unmanned guidance**Input:** $G=\left(N,E\right)$$—\mathrm{airport}\;\mathrm{surface}\;\mathrm{directed}\;\mathrm{graph};\;\mathrm{each}\;\mathrm{edge}\;e_{ij}$$\;\mathrm{has}\;\mathrm{length}\;d_{ij}$$,\;\mathrm{arc}\;\mathrm{length}\;l_{c}$;   U—set of assigned unmanned guidance;   Segment parameters (length d, $\mathrm{arc}\;\mathrm{length}\;l_{c}$, $\mathrm{entry}\;\mathrm{speed}\;v_{in}$, $\mathrm{exit}\;\mathrm{speed}\;v_{c}$, $\mathrm{maximum}\;\mathrm{speed}\;v_{\max}$, $\mathrm{acceleration}\;a_{cc}$, $\mathrm{deceleration}\;a_{dc}$);   Time-window and clearance parameters $\left(t_{buffer},\Delta t_{clearance}\right)$   Heuristic function and penalty weights of the improved A*. **Output:** $\left({p_{u}}^{*}\right)$—conflict-free trajectories with time windows.    1:  for each u∈U **do**    2:      invoke Algorithm 3 to generate the initial path of u    $3:\;\;\mathrm{compute}\;\mathrm{edge}\;\mathrm{travel}\;\mathrm{time}\;t\left(e_{ij}\right)$ using the speed-profile model    $4:\;\;\mathrm{recursively}\;\mathrm{obtain}\;\left\{t_{i}^{in},t_{i}^{out}\right\}$$\;\mathrm{to}\;\mathrm{form}\;\mathrm{the}\;\mathrm{initial}\;\mathrm{trajectory}\;\left\{p_{u}\right\}$    5:    **end for**    6: repeat    7:    invoke Conflict-Prediction Algorithm 4, Algorithm 5 and Algorithm 6    8:  collect conflict set C via event-scanning procedure    9:  if C≠∅ then    10:     invoke Algorithm 7    11:     **end if**    12:until C=∅


### 5.1. Optimization Objectives

The trajectory planning proposed in this study aims to achieve a dual-objective optimization of taxiing time and taxiing energy consumption for unmanned guidance tasks, while satisfying airport operational constraints and safety regulations [29].

Within a given time horizon T at the airport, a set of unmanned guidance vehicles $K=\left\{1,2,\ldots ,M\right\}$ is assigned to perform taxiing guidance operations for a set of arriving and departing flight guidance tasks $B=\left\{1,2,\ldots ,N\right\}$. The set of starting nodes for the guidance tasks is denoted as $P=\left\{1,2,\ldots ,n\right\}$, and the set of ending nodes as $D=\left\{1,2,\ldots ,m\right\}$. The time at which flight b (guided by unmanned guidance vehicle k) reaches any node i is denoted by $S_{k}^{i}\left(b\right)$.

1. For a single guidance task b∈B, if it is performed by an unmanned guidance vehicle k∈K, the total task duration can be expressed as $S_{K}^{D}(b)-S_{K}^{P}(b)$. For each individual guidance task, the optimization objective of the unmanned guidance vehicle trajectory planning is to minimize the maximum guidance duration, which can be formulated as
  (9) $$T(b){=\min}_{t}\max_{k\in K,b\in B}\left\{S_{K}^{D}(b)-S_{K}^{P}(b)\right\}=\min_{t}\max_{k\in K,b\in B}t_{k}^{i,j}(b)x_{k}^{i,j}(b)$$
  where $t_{k}^{i,j}(b)$ denotes the travel time of vehicle k∈K when performing guidance task b from node i to node j; $x_{k}^{i,j}(b)=1$ indicates that vehicle k∈K is assigned to perform the guidance task from node i to node j; otherwise, $x_{k}^{i,j}(b)=0$.
2. For a single guidance vehicle, if multiple guidance tasks forming a task chain $B^{\prime }=\left\{b_{1},b_{2},\ldots ,b_{q}\right\}$, the optimization objective for minimizing the task guidance duration is to minimize the total completion time of all tasks in the chain [30].
  (10) $$\begin{array}{l} T(b^{\prime })=\min_{t}\max_{k\in K,b\in B^{\prime }}\left\{\sum_{b\in B^{\prime }}\left(S_{K}^{D}\left(b\right)-S_{K}^{P}\left(b\right)\right)+\sum_{w=1,2,\ldots ,q-1}\Delta T_{w\left|w+1\right.}(k)\right\}\\ =\min_{t}\max_{k\in K,b\in B^{\prime }}\left\{\sum_{\left(i,j\right)\in A,b\in B^{\prime }}t_{k}^{i,j}\left(b\right)x_{k}^{i,j}\left(b\right)+\sum_{w=1,2,\ldots ,q-1}\Delta T_{w\left|w+1\right.}\right\} \end{array}$$
  where $\Delta T_{w\left|w+1\right.}$ denotes the travel time required between two consecutive guidance tasks. Each flight is associated with a single guidance task b∈B. Each guidance task is composed of three sequential phases, namely dispatch, guidance, and recovery.

The three phases described above can be represented as a tuple $b=\left(f,p,d,t,\varsigma \right)$. Therefore, when a guidance vehicle k∈K performs a guidance task b∈B, the total task duration equals the sum of the dispatch, guidance, and recovery phases.

(11) $$\left\{S_{K}^{D}\left(b\right)-S_{K}^{P}\left(b\right)\right\}=\sum_{\varsigma =1}^{3}\left(S_{k}^{\varsigma d}\left(b\right)-S_{k}^{\varsigma p}\left(b\right)\right)=\sum_{\varsigma =1}^{3}t_{k}^{\varsigma ,i,j}(b)x_{k}^{\varsigma ,i,j}\left(b\right)$$

where $\left(S_{k}^{\varsigma d}\left(b\right)-S_{k}^{\varsigma p}\left(b\right)\right)$ represents the duration of the current phase; $t_{k}^{\varsigma ,i,j}(b)$ denotes the travel time of vehicle k∈K when performing guidance task b during the current phase, from node i to node j.

3. For a single guidance task b∈B, the energy consumption of this task includes the energy consumed by the unmanned guidance vehicle and the guided aircraft forming the guidance unit. The optimization objective of unmanned guidance energy consumption is to minimize the maximum energy consumption during the guidance process.
  (12) $$E(b)=\min_{e}\max_{k\in K,b\in B}\sum_{\varsigma =1}^{3}E_{k}^{\varsigma d}\left(b\right)=\min_{t}\max_{k\in K,b\in B}\sum_{\varsigma =1}^{3}e_{k}^{\varsigma ,i,j}\left(b\right)x_{k}^{\varsigma ,i,j}\left(b\right)$$
  where $E_{k}^{\varsigma d}\left(b\right)$ represents the energy consumption of the vehicle k∈K when performing guidance task b during the current phase, and $e_{k}^{\varsigma ,i,j}\left(b\right)$ represents the energy consumption of the guidance task b from node i to node j during the current phase.

### 5.2. Constraints

4. During a single guidance task, each vertex in G can be visited only once.
  (13) $$\sum_{\left(i,j\right)\in E}x_{k}^{i,j}\left(b\right)=1,\forall j\in N\backslash \left\{0\right\}$$
  (14) $$\sum_{\left(i,j\right)\in E}x_{k}^{i,j}\left(b\right)=1,\forall i\in N\backslash \left\{n+m+1\right\}$$
5. In a single guidance task, the starting node i of each segment must be visited before its ending node j
  (15) $$S_{k}^{i}\left(b\right)\leq S_{k}^{j}\left(b\right),\mathrm{if}\;x_{k}^{i,j}\left(b\right)=1$$
  where $S_{k}^{i}\left(b\right)$ denotes the service start time at node i when performing task k; $S_{k}^{j}\left(b\right)$ denotes the service start time at node j when performing task k.
6. In a single guidance task, the start time of the ending node of each segment shall be at least equal to the start time of its starting node plus the travel time from node i to node j
  (16) $$M\left(1-x_{k}^{i,j}\left(b\right)\right)+S_{k}^{j}\left(b\right)\geq S_{k}^{i}\left(b\right)+t_{k}^{i,j}\left(b\right)$$
  where M is a sufficiently large constant.
7. The three phases of a guidance task are continuous, and the ending node of the current phase shall be the starting node of the next phase of the guidance task.
  (17) $$d_{\varsigma_{1}}=p_{\varsigma_{2}}$$
  (18) $$d_{\varsigma_{2}}=p_{\varsigma_{3}}$$
  where $d_{\varsigma_{1}}$ denotes the ending node of the dispatch phase, $p_{\varsigma_{2}}$ denotes the starting node of the guidance phase, $d_{\varsigma_{2}}$ denotes the ending node of the guidance phase, and $p_{\varsigma_{3}}$ denotes the starting node of the recovery phase.

## 6. Trajectory Planning Algorithm for Unmanned Guidance

The trajectory of unmanned guidance refers to the optimal conflict-free operational trajectory planned for an unmanned guidance vehicle or a guidance unit under the assigned task schedule and the topological constraints of the airport runway–taxiway system. The planning process consists of three stages: initial trajectory planning, conflict prediction, and conflict resolution

Specifically, in the initial trajectory planning stage, path planning is first performed through the heuristic function of the improved A* algorithm [31]. Subsequently, the planned path is combined with the speed profile algorithm to generate a high-precision speed profile, which is further used to construct the operational time windows and complete the generation of the initial trajectory for unmanned guidance. In the conflict prediction stage, potential conflicts are identified by calculating the overlap of time windows during which unmanned guidance vehicles and guidance units operate on the same segments. In the conflict resolution stage, the initial trajectories of unmanned guidance vehicles and guidance units are adjusted and optimized according to the conflict priority. Through this series of steps, the trajectory planning results for unmanned guidance can ultimately be generated to meet the task time requirements and the spatiotemporal safety constraints.

### 6.1. Initial Trajectory Planning for Unmanned Guidance

#### 6.1.1. Initial Trajectory Planning for Unmanned Guidance Based on the Improved A* Algorithm

In the trajectory planning of unmanned guidance, this study adopts an improved A* algorithm to generate the optimal trajectory for each task phase from its starting node to the corresponding ending node. The calculation procedure is described in Algorithm 3. The core idea of this algorithm is to design a heuristic function that estimates the cost for unmanned guidance vehicles and guidance units to move from the current node to the ending node of the current task phase, thereby guiding the search process toward the direction of minimum cumulative cost. Compared with the traditional A* algorithm, a constraint-based termination mechanism is introduced during node expansion and the evaluation function is reconstructed. A dynamically adjustable weighting coefficient ω is added before the heuristic function $h\left(n\right)$, and the improved evaluation function is expressed as follows:

(19) $$f\left(n\right)=g\left(n\right)+\omega h\left(n\right)$$

where $g\left(n\right)$ represents the actual path cost from the starting node P of the current phase to the current node n; $h\left(n\right)$ represents the heuristic estimated cost from the current node n to the ending node d of the current phase; ω is the dynamic weighting coefficient, and the Manhattan distance is adopted in this study to compute the heuristic value.

This improvement strategy adjusts the value of ω to effectively balance the weighting between the heuristic term and the actual cost term, so that the generated trajectory can achieve theoretical optimality while better satisfying the requirements of operational safety and feasibility on the airport surface.

Furthermore, when facing dynamic obstacles or multiple unmanned guidance conflicts, the improved A* algorithm can adaptively adjust ω according to the conflict prediction results. By modifying the evaluation function, unmanned guidance vehicles are guided to select alternative paths, thereby achieving conflict resolution and dynamic trajectory replanning for unmanned guidance [32]. The calculation of ω is given as follows:

(20) $$\omega =f_{reverse}+f_{turn}+f_{cross}+f_{conflict}+f_{surpass}$$

where $f_{reverse}$ represents the cost of reverse driving for unmanned guidance, $f_{turn}$ represents the cost of turning conflict for unmanned guidance, $f_{cross}$ represents the cost of crossing conflict for unmanned guidance, $f_{conflict}$ represents the cost of head-on conflict for unmanned guidance, and $f_{surpass}$ represents the cost of overtaking conflict for unmanned guidance. The conflict-related cost terms in the improved A* evaluation function are specified based on airport surface operating rules and taxiway network characteristics. The relative magnitudes of these conflict-related cost terms were finalized through repeated empirical tests under the airport surface operating rules, selecting values that reliably support conflict-triggered replanning while avoiding an overly restricted feasible search space.

#### 6.1.2. Time Window Calculation

**Figure 8:**
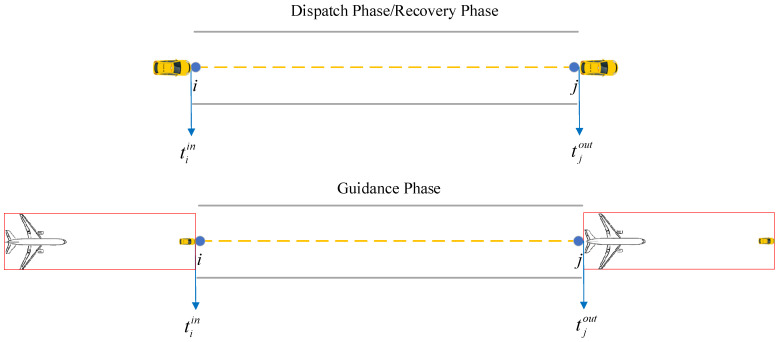
Time windows of unmanned guidance vehicles and guidance units.

Owing to the particularity of airport surface operations and the high safety requirements for unmanned guidance operations within the airside area, the concept of taxiing time windows is introduced to determine the entry and departure times of unmanned guidance vehicles and guidance units at each node n∈N and each segment e∈E [33]. The time window for unmanned guidance is illustrated in Figure 8.

The time $t_{i}^{in}$ at which the unmanned guidance enters segment $e_{ij}$ at node i is defined as the sum of the travel time over n segments and the buffer time required for transition between different types of segments [34].

(21) $$t_{i}^{in}=\sum_{i=1}^{n}t\left(e_{n}\right)+\left(1-\gamma \right)t_{buffer}$$

where $t\left(e_{n}\right)$ denotes the travel time of the unmanned guidance on the nth segment, $t_{buffer}$ represents the buffer time for the transition between different types of segments, which is determined based on the adopted speed profile and acceleration/deceleration constraints. The parameter γ=1 indicates that both the nth and n+1th segments are straight or belong to straight units; otherwise, γ=0.

The time $t_{i}^{out}$ at which the unmanned guidance leaves segment $e_{ij}$ at node i is defined as the sum of the entry time $t_{i}^{in}$ at the ith node and the time $\Delta t_{clearance}$ required for the unmanned guidance to travel from the front end to the rear end of the unmanned guidance when passing through the node.

(22) $$t_{i}^{out}=t_{i}^{in}+\Delta t_{clearance}$$

The travel time of the unmanned guidance along the nth segment is equal to the sum of the time spent on acceleration, constant speed, and deceleration.

(23) $$t\left(e_{n}\right)=t_{cc}\left(s\right)+t_{const}\left(s\right)+t_{dc}\left(s\right)$$

where $t_{cc}\left(s\right)$ denotes the travel time during acceleration, $t_{const}\left(s\right)$ denotes the travel time during constant-speed phase, and $t_{dc}\left(s\right)$ denotes the travel time during deceleration.

Real-time motion time calculation formula:

(24) $$t\left(s\right)=\left\{\begin{array}{cc} t_{ac}\left(s\right)=\frac{v_{s}-v_{in}}{a_{cc}}, & \mathrm{if}\;0\leq s<d_{1}\\ t_{const}\left(s\right)=\frac{d_{2}}{v_{s}}, & \mathrm{if}\;d_{1}\leq s<d_{2}\\ t_{dc}\left(s\right)=\frac{v_{s}-v_{c}}{a_{dc}}, & \mathrm{if}\;d_{2}\leq s\leq d_{3} \end{array}\right.$$

The algorithm is presented as follows (Algorithm 3):
**Algorithm 3** Improved A* algorithm for unmanned guidance vehicle trajectory planning**Input:** $G=\left(N,E\right)$$—\mathrm{airport}\;\mathrm{surface}\;\mathrm{directed}\;\mathrm{graph};\;\mathrm{start}\;\mathrm{and}\;\mathrm{goal}\;\mathrm{nodes}\;n_{s}$$\;\mathrm{and}\;n_{t}$;   $\mathrm{Segment}\;\mathrm{parameters}\;(\mathrm{maximum}\;\mathrm{speed}\;v_{\max}$$,\;\mathrm{acceleration}\;a_{cc}$$,\;\mathrm{deceleration}\;a_{dc}$);   $\mathrm{clearance}\;\mathrm{parameters}\;\left(t_{buffer},\Delta t_{clearance}\right)$;   Heuristic and penalty weights ω in **Formulas (19) and (20)** **Output:** Optimal path $n_{s}\to n_{t}$$\;\mathrm{with}\;\mathrm{entry}/\mathrm{exit}\;\mathrm{times}\;\left\{t_{i}^{in},t_{j}^{out}\right\}$.    $1:\;\;\mathrm{initialize}\;\mathrm{Open}\;\mathrm{and}\;\mathrm{Closed}\;\mathrm{sets};\;\mathrm{set}\;g\left(n_{s}\right)=0$    2:    while Open≠∅ do    $3:\;\;\;\;\mathrm{select}\;\mathrm{node}\;n_{i}$ with minimum **Formula (19)**    $4:\;\;\;\;\;\mathrm{for}\;\mathrm{each}\;\mathrm{adjacent}\;\mathrm{node}\;n_{j}$$\;\mathrm{of}\;n_{i}$ **do**    $5:\;\;\;\;\;\;\;\;\;\mathrm{compute}\;\mathrm{segment}\;\mathrm{time}\;t\left(e_{n}\right)$ using **Formula (22)**    $6:\;\;\;\;\;\;\;\;\;\mathrm{update}\;\mathrm{time}\;\mathrm{window}\;\left(t_{i}^{in},t_{j}^{out}\right)$ using **Formulas (19) and (20)**    7:          **if** time-window constraint violated then skip    $8:\;\;\;\;\;\;\;\;\;\mathrm{update}\;g\left(n_{i}\right)$$,\;f\left(n_{j}\right)$$\;\mathrm{and}\;\mathrm{record}\;parent\left(n_{j}\right)=n_{i}$    9:      **end for**    $10:\;\;\;\mathrm{move}\;n_{i}$ from Open to Closed    $11:\;\;\;\mathrm{if}\;n_{i}=n_{t}$ **then** break    12: **end while**    13: reconstruct and output the optimal trajectory P with $\;\mathrm{with}\;\left(t_{i}^{in},t_{j}^{out}\right)$

### 6.2. Conflict Prediction

**Figure 9:**
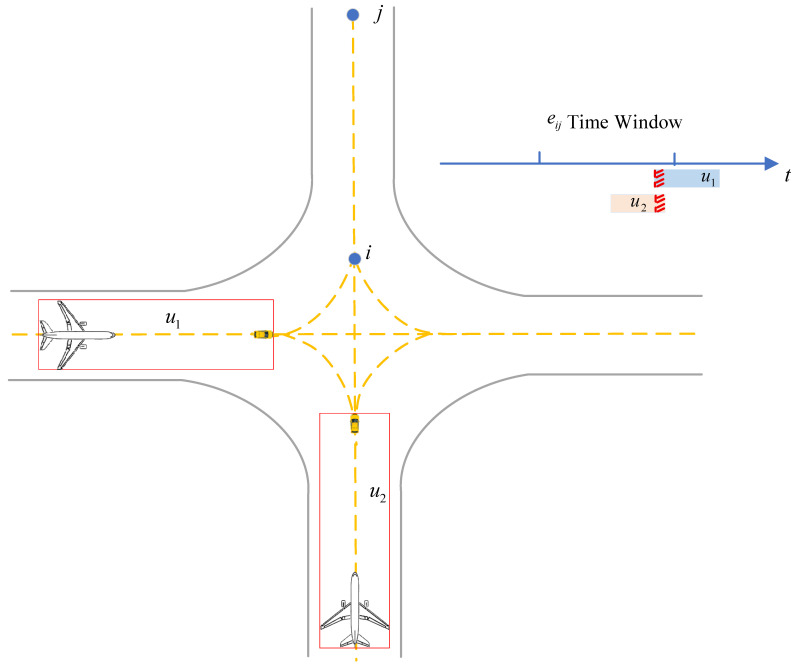
Illustration of crossing conflict and its time-window overlap determination.

Based on the topological traffic network of the airport surface runway–taxiway system, operational conflicts of unmanned guidance may occur at both nodes and segments. To ensure safe operations, three types of conflicts are defined as illustrated in Figure 9: crossing conflict, head-on conflict, and overtaking conflict. Where ς=1 represents the dispatch phase and ς=3 represents the recovery phase, during which the operating subject is the unmanned guidance vehicle; ς=2 represents the guidance phase, during which the operating subject is the unmanned guidance unit. Therefore, the operating subject bς of different phases within the same task is collectively referred to as the unmanned guidance set $U=\left\{1,2,\ldots ,u\right\}$.

1. Crossing Conflict

A crossing conflict refers to the situation in which two or more unmanned guidances operate on the same segment with overlapping time windows. The corresponding calculation method is presented in Algorithm 4.

As illustrated in Figure 9, the operating time of an unmanned guidance on segment $e_{ij}$ is defined as the time interval between entering node i and leaving node j, expressed as $T_{ij}^{u}=\left[t_{ij}^{u,i_{in}},t_{ij}^{u,j_{out}}\right]$. If the operating time interval $T_{ij}^{u}$ of two or more unmanned guidance on the same segment $e_{ij}$ overlap, a crossing conflict is identified.

(25) $$T_{ij}^{1}\cap T_{ij}^{2}\cap \ldots \cap T_{ij}^{n}\neq 0$$

where $t_{ij}^{u,i_{in}}$ denotes the time at which a guidance task enters segment $e_{ij}$ at node i, and $t_{ij}^{u,j_{out}}$ denotes the time at which a guidance task leaves segment $e_{ij}$ at node j.

The algorithm is presented as follows (Algorithm 4):
**Algorithm 4** Crossing-Conflict Prediction for Unmanned Guidance**Input:** $G=\left(N,E\right)$—airport surface directed graph;   U—set of assigned unmanned guidance;   $\mathrm{each}\;\mathrm{edge}\;e_{ij}\in E$$\;\mathrm{has}\;\mathrm{time}\;\mathrm{window}\;T_{ij}^{u}=\left[t_{ij}^{u,i_{in}},t_{ij}^{u,j_{out}}\right]$. **Output:** $c_{cross}$—set of crossing conflicts.    $1:\;\;c_{cross}\leftarrow \emptyset$    $2:\;\;\mathrm{for}\;\mathrm{each}\;\mathrm{edge}\;e_{ij}\in E$ **do**    $3:\;\;\;\;\;\mathrm{collect}\;\mathrm{all}\;\mathrm{occupancy}\;\mathrm{intervals}\;T_{ij}^{u}$$\;\mathrm{for}\;\mathrm{guidance}\;\mathrm{using}\;e_{ij}$    4:      sort enter/leave events by time ascending    5:     Active←∅    $6:\;\;\;\;\;\mathrm{for}\;\mathrm{each}\;\mathrm{event}\;\left(t,type,u\right)$ do    7:         if type=enter then insert u into Active    8:          else remove u from Active    $9:\;\;\;\;\;\;\;\;\;\mathrm{if}\;\left|Active\right|\geq 2$$\;\mathrm{then}\;\mathrm{record}\;\mathrm{conflict}\;\left[e_{ij},Active,\left(t_{i}^{in},t_{j}^{out}\right)\right]$$\;\mathrm{into}\;c_{cross}$
    10:     **end for**    11: **end for**    $12:\mathrm{return}\;c_{cross}$

2. Head-on Conflict

**Figure 10:**
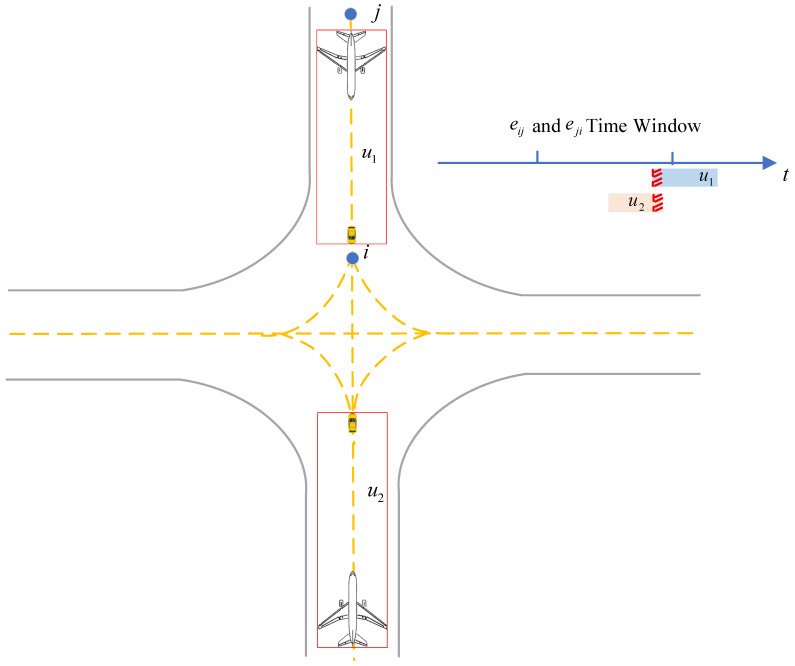
Illustration of head-on conflict and its time-window overlap determination.

A head-on conflict refers to the situation in which the same segment is simultaneously used by two or more unmanned guidances operating in opposite directions. The corresponding calculation method is presented in Algorithm 5. As illustrated in Figure 10, if the operating time $T_{ij}^{u}$ of two or more unmanned guidance on segment $e_{ij}$ overlaps with the operating time $T_{ji}^{u}$ on segment $e_{ji}$, a head-on conflict is identified.

(26) $$T_{ij}^{1}\cap T_{ji}^{2}\cap \ldots \cap T_{ij}^{n}\neq 0$$

The algorithm is presented as follows (Algorithm 5):
**Algorithm 5** Head-on conflict prediction for unmanned guidance**Input:** $G=\left(N,E\right)$—airport surface directed graph;   U—set of assigned unmanned guidance;   $\mathrm{each}\;\mathrm{edge}\;e_{ij}\in E$$\;\mathrm{has}\;\mathrm{time}\;\mathrm{window}\;T_{ij}^{u}=\left[t_{ij}^{u,i_{in}},t_{ij}^{u,j_{out}}\right]$. **Output:** $c_{head}$—set of head-on conflicts.    $1:\;\;c_{head}\leftarrow \emptyset$    $2:\;\;\mathrm{for}\;\mathrm{each}\;\mathrm{unordered}\;\mathrm{edge}\;pair\left\{i,j\right\}$$\;\mathrm{used}\;\mathrm{by}\;e_{ij}$$\;\mathrm{or}\;e_{ji}$ **do**    $3:\;\;\;\;\;\mathrm{collect}\;\mathrm{intervals}\;T_{ij}^{u}$$\;\mathrm{and}\;T_{ji}^{u}$    4:      sort events by time    $5:\;\;\;\;\;Active_{ij}\leftarrow \emptyset$$;\;Active_{ji}\leftarrow \emptyset$    $6:\;\;\;\;\;\;\mathrm{for}\;\mathrm{each}\;\mathrm{event}\;\left(t,type,u\right)$ **do**    $7:\;\;\;\;\;\;\;\;\;\mathrm{update}\;Active_{dir}$ according to enter/leave    $8:\;\;\;\;\;\;\;\;\;\;\;\;\;\;\;\;\mathrm{if}\;Active_{ij}\geq 1$$\;\mathrm{and}\;Active_{ji}\geq 1$$\;\mathrm{then}\;\mathrm{record}\;\mathrm{conflict}\;\left\{\left[\left(i,j\right),Active_{ij},Active_{ji},\left(t_{start},t_{end}\right)\right]\right\}$$\;\mathrm{into}\;c_{head}$    9:     **end for**    10: **end for**    $11:\mathrm{return}\;c_{head}$

3. Overtaking Conflict

**Figure 11:**
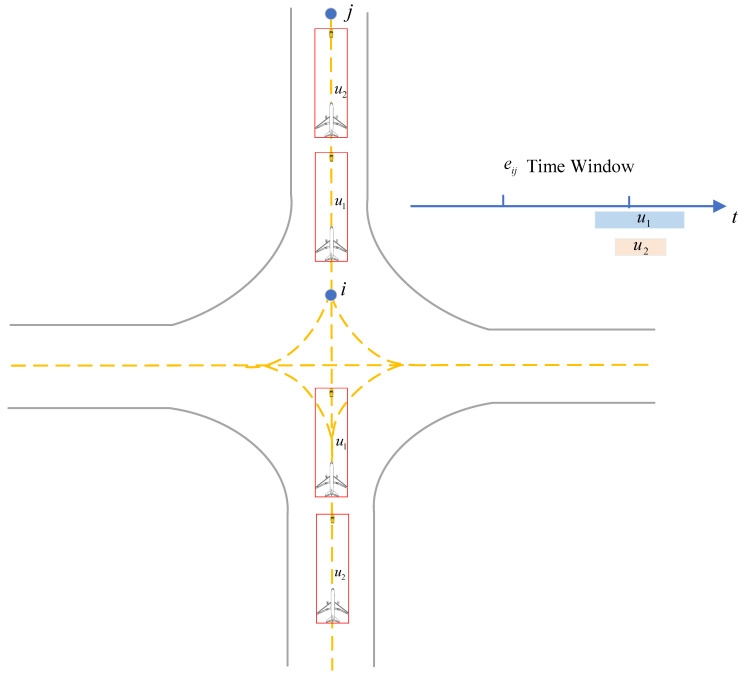
Illustration of overtaking conflict and its time-window overlap determination.

An overtaking conflict refers to the situation in which two or more unmanned guidances operate in the same direction on the same segment, and the following unmanned guidance overtakes the preceding one. The corresponding calculation method is presented in Algorithm 6. As illustrated in Figure 11, if the entry time $t_{ij}^{u,i_{in}}$ of one unmanned guidance entering segment $e_{ij}$ at node i is earlier than that of another unmanned guidance, but its leaving time $t_{ij}^{u,j_{out}}$ from node j is later than that of the other unmanned guidance, an overtaking conflict is identified.

(27) $$T_{ij}^{1}\cup T_{ij}^{2}\cup \ldots \cup T_{ij}^{n}=\max\left(T_{ij}^{1},T_{ij}^{2},\ldots ,T_{ij}^{n}\right)$$

The algorithm is presented as follows (Algorithm 6):
**Algorithm 6** Overtaking conflict detection for unmanned guidance**Input:** $G=\left(N,E\right)$—airport surface directed graph;   U—set of assigned unmanned guidance;   $\mathrm{each}\;\mathrm{edge}\;e_{ij}\in E$$\;\mathrm{has}\;\mathrm{time}\;\mathrm{window}\;T_{ij}^{u}=\left[t_{ij}^{u,i_{in}},t_{ij}^{u,j_{out}}\right]$. **Output:** $c_{overtake}$—set of overtaking conflicts.    $1:\;\;c_{overtake}\leftarrow \emptyset$    $2:\;\;\mathrm{for}\;\mathrm{each}\;\mathrm{directed}\;\mathrm{edge}\;e_{ij}\in E$ **do**    $3:\;\;\;\;\;\;\;\;\;\mathrm{collect}\;\mathrm{all}\;\mathrm{time}\;\mathrm{windows}\;T_{ij}^{u}$$\;\mathrm{for}\;\mathrm{guidance}\;\mathrm{using}\;e_{ij}$ in the same direction.    $4:\;\;\;\;\;\mathrm{find}\;\min t_{ij}^{u,i_{in}}$$\;\mathrm{and}\;\max t_{ij}^{u,j_{out}}$    $5:\;\;\;\;\;\mathrm{let}\;u^{*}$$\;\mathrm{be}\;\mathrm{the}\;\mathrm{guidance}\;\mathrm{satisfying}\;t_{ij}^{u^{*},i_{in}}=\min t_{ij}^{u,i_{in}}$$\;\mathrm{and}\;t_{ij}^{u^{*},j_{out}}=\max t_{ij}^{u,j_{out}}$    $6:\;\;\;\;\;\mathrm{for}\;\mathrm{each}\;v\in U\backslash u^{*}$ **do**    $7:\;\;\;\;\;\;\;\;\;\;\;\mathrm{if}\;t_{ij}^{u^{*},i_{in}}<t_{ij}^{u,i_{in}}$$\;\mathrm{and}\;t_{ij}^{u^{*},j_{out}}>t_{ij}^{u,j_{out}}$ **then**    $8:\;\;\;\;\;\;\;\;\;\;\;\;\;\;\;\;\mathrm{record}\;\left\{e_{ij},\left(u^{*},v\right),\left[t_{ij}^{v,i_{in}},t_{ij}^{v,j_{out}}\right]\right\}$$\;\mathrm{into}\;c_{overtake}$    9:           **end if**    10:     **end for**    11: **end for**    $12:\mathrm{return}\;c_{overtake}$


### 6.3. Conflict Resolution

Conflict resolution aims to generate conflict-free operational trajectories by adjusting the predicted conflicting trajectories of unmanned guidance. The corresponding calculation method is presented in Algorithm 7. Conflict resolution consists of conflict priority determination and trajectory adjustment strategies. First, the conflict priority determination follows the “first-come, first-served” principle. When two unmanned guidances are involved in conflict, the one with the earlier task start time maintains its original trajectory, while the one with the later start time needs to adjust its trajectory.

The expression for priority determination is given as follows:

(28) $$Priority\left(b_{1},b_{2},\ldots ,b_{n}\right)=\min\left(t_{\varsigma_{1}}^{b_{1}},t_{\varsigma_{1}}^{b_{2}},\ldots ,t_{\varsigma_{1}}^{bn}\right)$$

where $t_{\varsigma_{1}}^{bn}$ denotes the start time of guidance task $b_{n}$ in the first phase.

Second, in the trajectory adjustment stage, the heuristic weighting factor of the lower-priority unmanned guidance involved in the conflict is modified, so that the conflict constraint ω is activated in the evaluation function, thereby altering the path selection and generating a new feasible trajectory.

(29) $$\omega =\left\{\begin{array}{cc} f_{cross}^{b} & \;\mathrm{if}\;T_{ij}^{1}\cap T_{ij}^{2}\cap \ldots \cap T_{ij}^{n}\neq 0\\ f_{conflict}^{b} & \;\mathrm{if}\;T_{ij}^{1}\cap T_{ji}^{2}\cap \ldots \cap T_{ij}^{n}\neq 0\\ f_{surpass}^{b} & \mathrm{if}\;T_{ij}^{1}\cup T_{ij}^{2}\cup \ldots \cup T_{ij}^{n}=\max\left(T_{ij}^{1},T_{ij}^{2},\ldots ,T_{ij}^{n}\right) \end{array}\right.$$

The algorithm is presented as follows (Algorithm 7):
**Algorithm 7** Conflict resolution for unmanned guidance**Input:** $\left(P_{u}\right)$—initial trajectory set with edge-occupancy time $\mathrm{windows}\;T_{ij}^{u}=\left[t_{ij}^{u,i_{in}},t_{ij}^{u,j_{out}}\right]$ $c=\left\{c_{1},\ldots ,c_{m}\right\}$—conflict set; $\mathrm{each}\;c_{k}$$\;\mathrm{includes}\;\mathrm{conflict}\;\mathrm{type}\in \left\{\mathrm{crossing},\mathrm{head}-\mathrm{on},\mathrm{overtaking}\right\}$ $U_{k}$—conflict unmanned guidance; **Output:** $\left(P_{u}^{*}\right)$—conflict-free trajectory set. 1:  sort c by the start time in **Formula (28)** $2:\;\;\mathrm{for}\;\mathrm{each}\;\mathrm{conflict}\;c_{k}\in c$ **do** 3:        obtain unmanned guidance U_k_ and sort by **Formula (28)**: $\langle u_{1},u_{2},\ldots ,u_{k}\rangle$ $4:\;\;\;\;\;\;\mathrm{keep}\;u_{1}$ trajectory unchanged 5:        activate the corresponding conflict-penalty weight in **Formula (29)** $6:\;\;\;\;\;\;\mathrm{for}\;r=u_{k},\ldots ,2$ **do** $7:\;\;\;\;\;\;\;\;\;\;p_{u_{r}}$←**Formulas (20) and (21)** $8:\;\;\;\;\;\;\;\;\;\;\mathrm{recompute}\;T_{ij}^{u}=\left[t_{ij}^{u,i_{in}},t_{ij}^{u,j_{out}}\right]$$\;\mathrm{and}\;\mathrm{update}\;\left(p_{u}\right)$ 9:        **end for** $10:\;\;\;\;\;\mathrm{if}\;c_{k}$ is resolved then remove it from c 11:  **end for** 12:repeat Steps 2–11 until c=∅ $13:\mathrm{return}\;\left(p_{u}^{*}\right)$


Through the above process, each unmanned guidance can generate a conflict-free operating trajectory for every phase of its assigned task, thereby forming a complete unmanned guidance trajectory planning scheme.

Although this study does not conduct dedicated robustness experiments under special or extreme operational scenarios, the proposed trajectory planning framework inherently supports dynamic operational changes through its time-window-based conflict prediction and priority-driven trajectory replanning strategy. When operational information is updated, such as flight delay/early arrival or incremental guidance task insertion, the start time of the corresponding guidance task is updated, and the time-window calculation procedure is re-executed to update the temporal occupancy of trajectory segments and nodes. Based on the updated time-window constraints, potential crossing, head-on, and overtaking conflicts with existing active trajectories are re-identified. If potential conflicts are detected, trajectory adjustment is subsequently performed within the proposed planning framework to generate an updated feasible trajectory, thereby completing the trajectory planning process under dynamic operational conditions.

## 7. Case Study

### 7.1. Case Design

**Figure 12:**
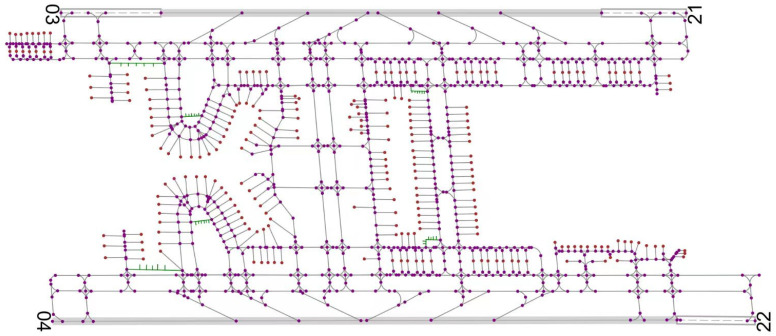
Network model of a major airport in Southwest China.

The case study is conducted using a major airport in Southwest China as the background, and the corresponding model parameters are configured as shown in Table 1. Flight operation data from a typical working day at this airport were collected, as summarized in Table 2. A total of 357 flights are included, consisting of 107 arriving flights and 250 departing flights. Figure 12 illustrates the runway–taxiway system traffic network of the airport, which comprises runways, taxiways, parking stands, and parking areas. The airport contains two parallel runways, 235 parking stands, and five dedicated parking areas for unmanned guidance vehicles.

### 7.2. Analysis of Guidance Time Results

The computational cost is primarily influenced by the number of nodes expanded during the improved A* search and the frequency of conflict-triggered replanning, both of which may increase with traffic density. In the full-day experiment involving 357 flight movements, the proposed framework remains computationally tractable and is able to generate conflict-free operational trajectories for all flights.

For the corresponding flights, taxiing trajectories based on unmanned guidance vehicles are designed, and the phase times after introducing unmanned guidance are summarized in Table 3.

For arriving flights at this airport, the unmanned guidance operation shows an average dispatch phase time of 3.105 min, an average guidance phase time of 5.493 min, and an average recovery phase time of 4.438 min. Compared with autonomous taxiing without the use of unmanned guidance, arriving flights achieve an average taxiing-time reduction of 7.729 min when assisted by unmanned guidance, corresponding to a 58.46% improvement in operational efficiency. For departing flights using unmanned guidance, the unmanned guidance operation shows an average dispatch phase time of 2.720 min, an average guidance phase time of 10.0741 min, and the average recovery phase time is 4.438 min; with the introduction of unmanned guidance, the average taxiing time is reduced by 4.084 min, corresponding to a 43.65% improvement in operational efficiency. These results indicate that the improved A* algorithm proposed in this study generates guidance phase taxiing trajectories in a more systematic manner, enabling more efficient utilization of airport taxiway resources and thereby improving operational efficiency.

**Figure 13:**
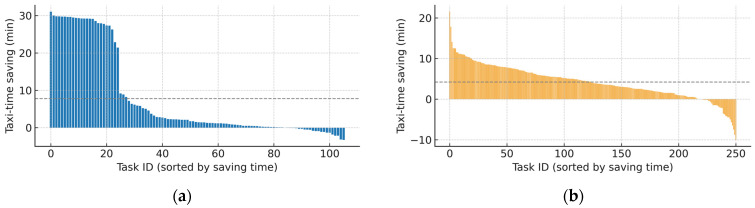
Distribution of taxiing time savings during the guidance phase: (**a**) represents distribution of taxiing time savings for arriving flights; (**b**) represents distribution of taxiing time savings for departing flights.

**Figure 14:**
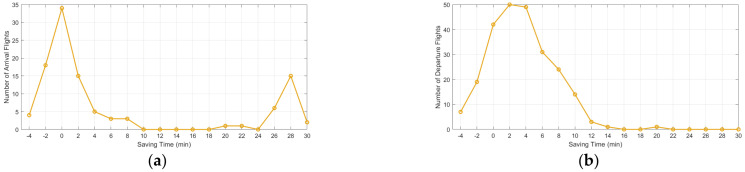
Variation trends of taxiing-time optimization during the guidance phase: (**a**) represents the optimization time variation trends for arriving flights; (**b**) represents the optimization time variation trends for departing flights.

The taxiing time savings of individual arriving and departing flights during the guidance phase are illustrated in Figure 13a,b. The corresponding distributions of taxiing time savings for arriving and departing flights are analyzed in Figure 14a,b. The results show that the taxiing time of the vast majority of flights is significantly reduced, and for both arriving and departing flights, most taxiing time savings fall within 4 min (45.8% for arriving flights and 36.8% for departing flights). Most tasks exhibit a clear positive optimization effect, with only 22 tasks showing negative operational optimization time among the arriving flights and only 32 tasks showing negative operational optimization time among the departing flights. The distribution of taxiing-time savings for arriving flights exhibits a “high at both ends with a gradual decline in the middle” pattern, where negative optimization (−4 to 0 min) and substantial optimization (26–30 min) appear at the two ends, while the middle part shows a decreasing trend as the magnitude of time savings increases. This distribution is consistent with the characteristics of actual airport surface operations: under the global optimization-oriented trajectory planning strategy adopted in this study, some tasks execute rerouting maneuvers during spatiotemporal conflict prediction and resolution in order to ensure the feasibility of the overall solution and to enhance global operational efficiency, which may result in a slight increase in the optimized taxiing time. Such negative optimization values are normal outcomes in the global optimization process and reflect the characteristic of “local concessions made to achieve global optimality.” Under the conventional Follow-Me guidance mode, some arriving flights may experience relatively long taxiing time due to factors such as extended route choices, additional detour segments, and discontinuous speed adjustments. The speed profile model and trajectory planning method proposed in this study can significantly reduce unnecessary taxi distances and avoidable speed losses, thereby yielding substantial time savings and naturally leading to the observed bimodal distribution [38].

These negative optimization cases mainly arise from local trajectory adjustments during spatiotemporal conflict prediction and resolution. When potential conflicts are identified, the proposed method adaptively adjusts the dynamic weighting coefficient in the improved A* evaluation function, thereby modifying the search cost and causing the replanned trajectory to deviate from the original shortest-time path by adopting alternative local routes to satisfy global feasibility and spatiotemporal safety constraints. Consequently, a small number of tasks may experience slightly increased travel distance and taxiing time, which reflects the global optimization characteristic of “local concessions made to achieve global optimality.”

The distribution of taxiing-time savings for departing flights exhibits a “left-skewed rise with a gradual decline in the middle” pattern; that is, negative optimization (−4 to 0 min) appears on the left side, while the middle part shows a decreasing trend as the magnitude of time savings increases. This distribution pattern is likewise consistent with the operational characteristics of actual airport surface environments. Under the global optimization-oriented trajectory planning strategy adopted in this study, certain tasks must perform rerouting in local segments during spatiotemporal conflict prediction and resolution to ensure the feasibility of the global solution and maintain the temporal stability of departing taxiing trajectories, thereby resulting in slight negative optimization. Such negative values are normal outcomes within the global optimization process and likewise reflect the scheduling characteristic of “local concessions made to achieve global optimality.” The optimization patterns observed for both arriving and departing flights further demonstrate the applicability and effectiveness of the proposed trajectory planning model under different operational conditions.

**Figure 15:**
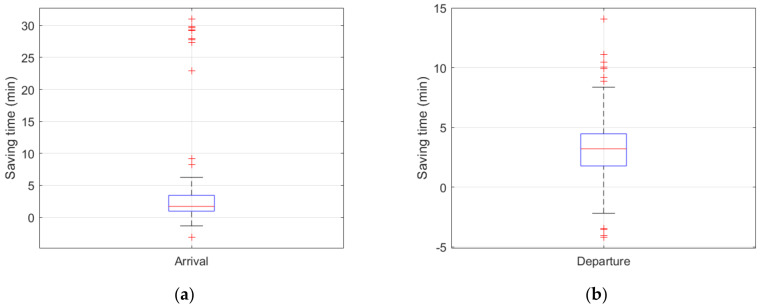
Distribution of taxiing-time optimization during the guidance phase: (**a**) represents the optimization-time distribution for arriving flights; (**b**) represents the optimization-time distribution for departing flights. In the box plot, the box represents the interquartile range, the horizontal line inside the box indicates the median, the whiskers denote the non-outlier range, and the red “+” symbols represent outliers.

To analyze the time-optimization performance of arrival guidance tasks, Figure 15a presents the distribution of taxiing-time savings for the arriving flights. Among them, most tasks exhibit taxiing-time savings concentrated within a relatively small range, with limited overall fluctuation. The improved A* trajectory-planning algorithm reduces unnecessary detours, and the speed-profile model enhances the continuity of taxiing speeds, thereby enabling arrival guidance tasks to achieve more consistent time improvements. Figure 15b presents the distribution of taxiing-time savings for the departure guidance tasks, which is used to evaluate the optimization performance of the model under departure scenarios.

The taxiing time savings exhibit a relatively wide distribution range, and some tasks achieve substantial reductions in taxiing time, indicating that departure taxiing possesses greater potential for optimization. The improved A* trajectory planning algorithm generates lower-cost taxiing paths within the complex taxiway network, while the speed-profile model provides more pronounced optimization benefits over long-distance taxiing. In addition, the conflict-prediction and time-window adjustment strategies effectively reduce queueing delays, thereby yielding sizable taxiing-time savings.

To further interpret the above energy-saving results from a system-level perspective, the reported 34.52% energy reduction corresponds to the guidance-phase energy saving of the guided unit consisting of the unmanned guidance vehicle and the guided aircraft. The total energy consumption therefore includes both the operational energy of the unmanned guidance vehicle and the taxiing energy of the aircraft. In practical airport operations, aircraft taxiing is conducted under low-speed and tightly regulated conditions. Under such comparable operating conditions, taxiing energy consumption is approximately proportional to taxiing distance, making trajectory shortening a primary determinant of total energy expenditure. Moreover, for the same taxiing distance, the absolute energy consumption of an aircraft is significantly higher than that of an unmanned guidance vehicle, due to its substantially larger propulsion power demand. As a result, the energy saving achieved through taxiing distance reduction is mainly reflected in the aircraft component, while the contribution of the unmanned guidance vehicle remains relatively small.

In addition to trajectory optimization, the acceleration–cruise–deceleration speed-profile design further contributes to energy efficiency by maintaining a smoother and more stable taxiing motion, thereby reducing unnecessary speed fluctuations and traction losses, particularly during acceleration and deceleration. Compared with trajectory shortening, this effect plays a secondary but complementary role in the overall energy-saving performance, and the combined effect of these two mechanisms leads to the observed reduction in guidance-phase energy consumption.

As shown in Table 4, all conflicts are successfully resolved in both normal and peak-congestion scenarios. Although the average time reduction decreases from 43.65% to 27.26% under peak traffic conditions, the optimization performance remains significant. This indicates that the proposed framework does not exhibit abrupt performance degradation when traffic density increases, demonstrating its robustness under stressed operating conditions.

### 7.3. Energy Consumption Analysis

The energy consumption of the surface taxiing guidance process after introducing unmanned guidance vehicles is analyzed. During the dispatch and recovery phases, the energy consumption of the unmanned guidance vehicle is approximately 0.9–1.1 kWh/km when converted from the fuel consumption level of conventional Follow-Me vehicles commonly used at domestic airports (gasoline SUVs with a fuel consumption of 10–12 L/100 km) [39]. In contrast, the unmanned guidance vehicle, which is built on a low-speed electric vehicle platform, consumes only about 0.06–0.10 kWh/km during surface operations. Based on typical task mileage, the energy consumption ratio between conventional Follow-Me vehicles and unmanned guidance vehicles is approximately 1:0.3, corresponding to an energy-saving rate of about 70% [40].

**Figure 16:**
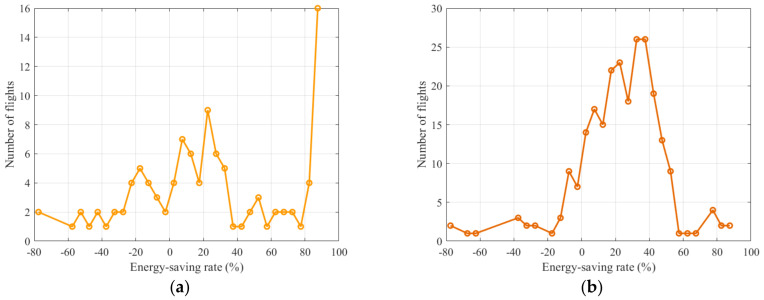
Energy optimization efficiency of individual tasks during the guidance phase: (**a**) represents the energy optimization distribution for guidance units of arriving flights; (**b**) represents the energy optimization distribution for guidance units of departing flights.

For the guidance phase, considering the effects of different aircraft types (e.g., A320, A330, ERJ190), the energy consumption of the guidance unit consists of two components: the aircraft taxiing energy consumption and the energy consumption of the unmanned guidance vehicle [41]. The energy optimization efficiencies of the arriving and departing guidance units are shown in Figure 16a and Figure 16b, respectively, indicating that most guidance units achieve substantial energy savings during the taxiing process. A small number of guidance units exhibit negative energy-saving efficiency. The energy-optimization efficiency of arriving flights exhibits a distribution characterized by predominantly positive energy savings, a small proportion of negative values, and a clearly identifiable high efficiency peak. The improved A* trajectory-planning algorithm prioritizes ensuring the feasibility of the global solution and therefore adjusts a small number of individual tasks when necessary. The energy-optimization efficiency of departing flights exhibits a pattern characterized by moderate energy savings as the dominant outcome, a stable overall distribution, and very few negative values. By means of well-designed speed profiles and the reasonable selection of operational trajectories based on the improved A* trajectory-planning algorithm, a stable energy-saving performance is achieved.

**Figure 17:**
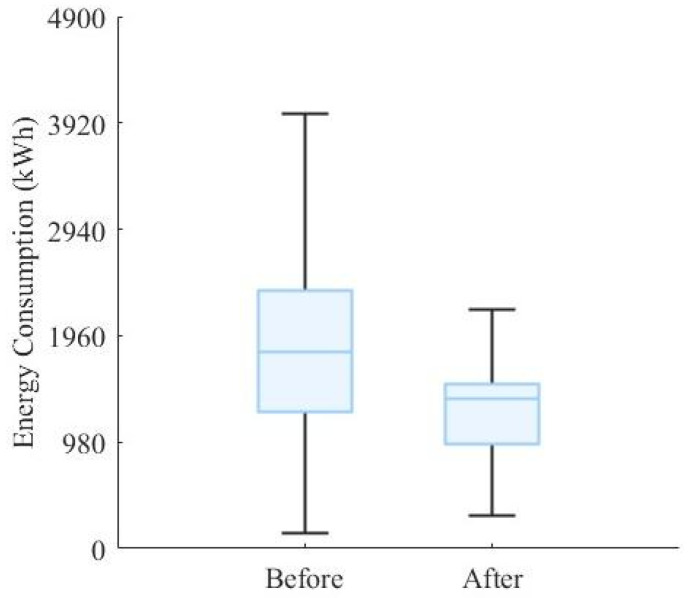
Total energy-optimization performance of unmanned guidance.

**Figure 18:**
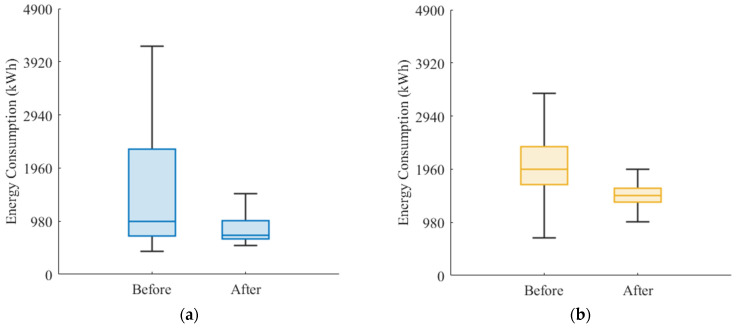
Energy optimization performance of guidance units for arriving and departing flights: (**a**) represents the guidance unit energy optimization during the guidance phase for arriving flights; (**b**) represents the guidance unit energy optimization during the guidance phase for departing flights.

At this stage, most arriving flight guidance units achieve energy optimization levels between 10% and 60%, whereas the departing flight guidance units achieve energy savings in the range of 20% to 40%. Furthermore, the total energy-optimization performance of the entire unmanned guidance process is analyzed. As shown in Figure 17, the overall energy consumption decreases by approximately 37% on average when using the proposed algorithm, while the interquartile range is significantly reduced. This optimization approach not only lowers the average energy consumption level but also enhances the stability of the energy distribution profile. For the energy consumption of arriving flight tasks, the optimization results shown in Figure 18a indicate that the pre-optimization energy distribution profile is highly dispersed. After introducing the guidance unit, the overall distribution shifts downward, with the median decreasing by approximately 40% and the upper and lower bounds becoming more concentrated [42]. For the energy consumption of departing flight tasks, as shown in Figure 18b, the distribution is relatively compact, and the average energy-saving rate reaches 35–40%. It can be observed that, because departing taxiing in this study is dominated by acceleration and constant-speed segments, the speed-profile model combined with the improved A* trajectory-planning algorithm effectively reduces thrust fluctuations and braking losses, thereby further enhancing overall energy efficiency [43].

## 8. Conclusions

1. For the problem of unmanned guidance trajectory planning and energy optimization on the airport surface, a speed profile model and an energy consumption model for unmanned guidance are proposed, enabling the joint optimization of both aspects; By incorporating the actual operational rules of unmanned guidance on the airport surface, the improved A* algorithm is employed to digitalize these operational constraints. Considering the three types of conflicts that may occur during unmanned guidance operations on the airport surface, conflicts are identified through time-window determination; furthermore, a task-priority-based conflict-resolution algorithm is designed.
2. A case study is conducted based on a major airport in Southwest China. The results indicate that the improved A* trajectory planning algorithm substantially enhances the operational efficiency of airport surface guidance. With the introduction of unmanned guidance vehicles and the adoption of the proposed algorithm, guided taxiing effectively reduces both taxiing time and energy consumption, with 79% of arriving flights and 87% of departing flights achieving reductions in operational time. In terms of airport surface energy consumption, 71% of arriving flights and 85% of departing flights achieve reductions in energy consumption during the guidance phase. Overall, with the application of the proposed unmanned guidance trajectory-planning model, the total operational efficiency increases by 43.65%, and the total energy consumption during the guidance phase is reduced by 34.52%, thereby achieving coordinated optimization of operational efficiency and energy consumption.
3. Future research may investigate trajectory planning strategies for scenarios in which flights experience delays or unmanned guidance vehicles encounter unexpected events, in order to enhance the robustness of the proposed algorithms and models.

## Figures and Tables

**Table 1:** Parameters of the trajectory planning and energy consumption models (default values and recommended ranges).

Symbol	Meaning	Unit	Default Value	Recommended Range	Description	Source
$f_{reverse}$	Weight of reverse-driving conflict cost	—	100	80–150	Penalty for reversing or wrong-way driving paths	Model assumption
$f_{turn}$	Weight of turning conflict cost	—	20	10–30	Penalty for frequent sharp turns or continuous turning	Model assumption
$f_{cross}$	Weight of crossing conflict cost	—	20	10–30	Penalty to discourage paths passing through busy intersections	Model assumption
$f_{conflict}$	Weight of head-on conflict cost	—	40	30–60	Increased penalty for head-on lane-occupying paths	Model assumption
$f_{surpass}$	Weight of overtaking conflict cost	—	20	10–30	Penalty to prevent same-direction overtaking and maintain a safety following distance	Model assumption
$t_{buffer}$	Buffer time between nodes/segments	s	5	3–10	Safety buffer for time-window propagation	Derived from speed profile and motion constraints
$\Delta t_{clearance}$	Departure time of the unmanned guidance vehicle from a node	s	15	—	Used for time-window calculation	Computed value
$m^{v}$	Equivalent mass of the unmanned guidance vehicle	kg	1500	1200–1800	Constant term used in the kinetic-energy component	Typical engineering value
g	Gravitational acceleration	m·s^−2^	9.81	—	Constant	Standard constant
$c_{r}^{v}$	Rolling resistance coefficient of the unmanned guidance vehicle	—	0.015	0.01–0.02	Smooth asphalt/cement pavement	Literature-based [35]
$c_{r}^{f}$	Rolling resistance coefficient of the Follow-Me vehicle	—	0.018	0.016–0.020	Smooth asphalt/cement pavement	Literature-based [35]
ρ	Air density	kg·m^−3^	1.225	1.1–1.3	Sea level, 15 °C	Standard constant
$C_{d}A^{v}$	Aerodynamic drag coefficient x frontal area of the unmanned guidance vehicle	m^2^	0.80	0.7–1.2	Determined by vehicle body shape	Literature-based [36]
$C_{d}A^{f}$	Aerodynamic drag coefficient x frontal area of the Follow-Me vehicle	m^2^	0.90	0.80–0.95	Determined by vehicle body shape	Literature-based [36]
$\eta^{v}$	Transmission efficiency of the unmanned guidance vehicle	—	0.90	0.85–0.95	Electric drivetrain efficiency	Typical engineering value
$\eta^{f}$	Transmission efficiency of the Follow-Me vehicle	—	0.25	0.20–0.30	Fuel drivetrain efficiency	Typical engineering value
LHV	Lower heating value of aviation kerosene	MJ·kg^−1^	43.0	42.8–43.3	Used to convert fuel mass flow rate into energy consumption	Standard fuel property
${\dot{m}}_{taxi}^{Narrow}$	Fuel burn rate during taxiing (narrow-body aircraft)	kg·s^−1^	0.195	0.15–0.25	Narrowbody	Literature-based [37]
${\dot{m}}_{taxi}^{Wide}$	Fuel burn rate during taxiing (wide-body aircraft)	kg·s^−1^	0.430	0.35–0.50	Widebody	Literature-based [37]
${\dot{m}}_{taxi}^{Region}$	Fuel burn rate during taxiing (regional aircraft)	kg·s^−1^	0.137	0.10–0.18	Regional	Literature-based [37]

Note: The default values in the table are used for the standard case study, while the ranges are applied for sensitivity analysis. The physical parameters correspond to the energy consumption model, and the weight parameters are consistent with those used in the conflict cost function.

**Table 2:** Arrival and departure flight data.

Flight Number	Arrival/Departure Type	Arrival/Departure Time	Stand Arrival/Pushback Time	Runway Used	Aircraft Type	Stand Used
HO1017	Arrival	00:03	00:11	22	A321	142
GJ8872	Arrival	00:08	00:19	22	3NEO	168
KY8348	Arrival	00:00	00:25	22	B737	159
…	…	…	…	…	…	…
BK2898	Departure	23:19	23:30	21	B738	156
MU9951	Departure	23:21	23:33	22	B738	123
CF9017	Departure	23:59	00:12	22	B738	724

**Table 3:** Time consumption of each phase of unmanned guidance.

	Phase Type	Dispatch Phase	Guidance Phase	Recovery Phase
Arrival/Departure				
Arrival		332.2574 min (5.54 h)	587.7280 min (9.80 h)	474.8694 min (7.914 h)
Departure		679.9318 min (11.33 h)	2518.5314 min (41.98 h)	1716.8740 min (28.614 h)
Total		1012.1892 min (16.87 h)	3106.2631 min (51.78 h)	2191.7434 min (36.53 h)

**Table 4:** Robustness evaluation under normal and peak-congestion operating scenarios.

Scenario	Number of Tasks	Conflict Resolution Success Rate	Average Operation Time Reduction (%)
Normal (full day)	357	100%	43.65%
Peak (11:00–12:00)	27	100%	27.26%

## Data Availability

The authors confirm that the data supporting the findings of this study are available within the article. The data presented in this study are available on request from the corresponding author due to privacy.

## Appendix A

**Table A1:** Network structure and sets.

Symbol	Description	Unit
$G_{X}$	The runway–taxiway system network	—
$N_{R}$	Set of runway entry, runway exit, and aircraft stand nodes	—
$N_{V}$	Set of parking positions	—
$N_{W}$	Set of waiting points near stands or runway exits	—
$N_{X}$	Set of taxiway intersections	—
$E_{X}$	Set of directed edges	—
$E_{X}^{R}$	Set of runway edges	—
$E_{X}^{V}$	Set of taxiway edges	—
$E_{X}^{W}$	Set of stand- and waiting-point edges	—

**Table A2:** Flights and guidance tasks.

Symbol	Description	Unit
F	set of flights considered in the guidance operation	—
B	set of guidance tasks	—
$B^{arr}$	set of guidance tasks for arriving aircraft	—
$B^{dep}$	set of guidance tasks for departing aircraft	—
U	unmanned guidance (unmanned guidance vehicle and guidance unit)	—
K	set of unmanned guidance vehicles	—
ς	operation phase indicator (ς=1,2,3 for dispatch, guidance, and recovery)	—

**Table A3:** Trajectory attribute mappings and time variables.

Symbol	Description	Unit
$p:I\to N_{R}\cup N_{V}\cup N_{W}$	The starting node of the current phase	—
$d:I\to N_{R}\cup N_{V}\cup N_{W}$	The ending node of the current phase	—
T	Time domain	—
t:I→T	The starting time of the current phase	s

**Table A4:** Network attributes and operational parameters.

Symbol	Description	Unit
$L_{X}$	The length of each edge in the network	m
$v_{X}$	The operating taxi speed adopted on each edge	$m\cdot s^{-1}$

**Table A5:** Guidance task representation.

Symbol	Description	Unit
$b=\left(f,p,d,t,\varsigma \right)$	Tuple representation of a guidance task	—
